# Overall survival based on oncologist density in the United States: A retrospective cohort study

**DOI:** 10.1371/journal.pone.0250894

**Published:** 2021-05-12

**Authors:** Sudeep K. Siddappa Malleshappa, Smith Giri, Smit Patel, Tapan Mehta, Leonard Appleman, Scott F. Huntington, Vida Passero, Rahul A. Parikh, Kathan D. Mehta

**Affiliations:** 1 Division of Hematology-Oncology, Department of Medicine, UMass-Baystate Medical Center, Springfield, MA, United States of America; 2 Division of Hematology-Oncology, Department of Medicine, University of Birmingham, Birmingham, AL, United States of America; 3 Department of Neurology, University of Connecticut, Farmington, CT, United States of America; 4 Department of Neurology, University of Minnesota, Minneapolis, MN, United States of America; 5 Division of Hematology-Oncology, Department of Medicine, University of Pittsburgh Medical Center, Pittsburgh, PA, United States of America; 6 Division of Hematology-Oncology, Department of Medicine, Yale School of Medicine, New Haven, CT, United States of America; 7 Division of Hematology-Oncology, Department of Medicine, University of Kansas Medical Center, Kansas City, KS, United States of America; The Cancer Institute of New Jersey, Robert Wood Johnson Medical School, UNITED STATES

## Abstract

Medically underserved areas (MUA) or health professional shortage areas (HPSA) designations are based on primary care health services availability. These designations are used in recruiting international medical graduates (IMGs) trained in primary care or subspecialty (e.g., oncology) to areas of need. Whether the MUA/HPSA designation correlates with Oncologist Density (OD) and supports IMG oncologists’ recruitment to areas of need is unknown. We evaluated the concordance of OD with the designation of MUAs/HPSAs and evaluated the impact of OD and MUA/HPSA status on overall survival. We conducted a retrospective cohort study of patients diagnosed with hematological malignancies or metastatic solid tumors in 2011 from the Surveillance Epidemiology and End Results (SEER) database. SEER was linked to the American Medical Association Masterfile to calculate OD, defined as the number of oncologists per 100,000 population at the county level. We calculated the proportion of counties with MUA or HPSA designation for each OD category. Overall survival was estimated using the Kaplan-Meier method and compared between the OD category using a log-rank test. We identified 68,699 adult patients with hematologic malignancies or metastatic solid cancers in 609 counties. The proportion of MUA/HPSA designation was similar across counties categorized by OD (93.2%, 95.4%, 90.3%, and 91.7% in counties with <2.9, 2.9–6.5, 6.5–8.4 and >8.4 oncologists per 100K population, p = 0.7). Patients’ median survival in counties with the lowest OD was significantly lower compared to counties with the highest OD (8 vs. 11 months, p<0.0001). The difference remained statistically significant in multivariate and subgroup analysis. MUA/HPSA status was not associated with survival (HR 1.03, 95%CI 0.97–1.09, p = 0.3). MUA/HPSA designation based on primary care services is not concordant with OD. Patients in counties with lower OD correlated with inferior survival. Federal programs designed to recruit physicians in high-need areas should consider the availability of health care services beyond primary care.

## Introduction

The American Association of Medical Colleges (AAMC) recent report predicts a shortfall of between 42,600 and 121,300 physicians, including 33,800 to 72,700 non-primary care physicians by 2030 [1]. American Society of Clinical Oncology (ASCO) has predicted a shortage of 1,521 oncologists by 2025 [2]. While there is some disagreement about whether physician shortage exists in the United States [3], there is consensus that the distribution of physicians in the United States is more concentrated in major metropolitan areas, creating pockets of underserved areas [4–6]. It was noted that the 5-year relative survival rate is lower in rural areas compared to metropolitan areas, even though cancer incidence is found to be lower in rural areas [7]. One strategy to reduce this disparity is requiring non-US citizen international medical graduates (IMGs) to work in underserved areas [8].

Every year at least 3500 non-US citizen IMGs match into a residency or fellowship program using a J1 visa [9]. Under the traditional pathway, J1 visas require the trainee to return to their home country for at least two years upon completion of medical training. The Conrad-30 program was established to allow state departments of health to sponsor a waiver of the home residency requirement in return for the IMGs service in medically underserved areas (MUAs) or health professional shortage areas (HPSAs) [8]. Each state has thirty waivers available, and priority is generally given to primary care providers (PCPs) [10,11]. The designation of MUA or HPSA is based on a shortage of PCPs, percent of the population below the federal poverty level, infant health, and travel time to the nearest source of care; but it does not consider disparities in specialty care such as medical oncology [12,13].

AAMC’s previous report on physician shortage calls for additional research in specific specialties such as cardiology and oncology, where disease burden has increased in recent times [14]. It is unclear if MUA or HPSA designation is concordant with the density of oncologists. While the Conrad-30 program allows states to have IMG specialists (e.g., oncologists) on a J1 visa to work in MUAs or HPSAs, it is unknown if there are a higher number of oncologists working on a visa in areas with a lower density of oncologists. Furthermore, the impact of oncologist density (OD) on overall survival has not been studied in patients with metastatic solid cancer and hematologic malignancies.

## Materials and methods

### Data sources

We merged Surveillance, Epidemiology, and End Results (SEER) 1973–2014 Research Plus Additional Custom Treatment data [3] with American Medical Associations (AMA) physician’s master file for the year 2011. Linkage was at the county (or county equivalent) level using Federal Information Processing Standards (FIPS) codes. SEER data contains information on patient demographics, stage at diagnoses, and survival information. Out of 612 FIPS code areas present in SEER during 2011, 3 were excluded due to unknown FIPS county code. The AMA physician master file contains information on physicians’ demographics, practice location, specialty information, and visa status. Oncologists who were in training or have retired were excluded. MUA or HPSA designation for the year 2011 was obtained from Health Resources and Service Administration and merged to the data file by FIPS code. We accessed and analyzed these data by signing data use agreement in compliance with the Health Insurance Portability and Accountability Act of 1996, so this protocol is exempted from the institutional review board’s approval at the University of Kansas Medical Center.

### Patient selection

We included patients with age greater than 19 years who had newly diagnosed hematologic malignancies or metastatic solid cancers in the year 2011. Patients with CNS cancers, patients with CNS metastasis, and patients who required major surgery as a part of primary treatment were excluded. Follow-up information, including vital status and survival information, was available through 2014. The study did not involve any human participant and was deemed exempt from the institutional review board.

### Variables

Our primary explanatory variable was OD, defined as the number of oncologists per 100,000 adult population in the FIPS code area. The number of people with age greater than 19 living in the given FIPS code area in 2011 was identified using SEER population files and used as a denominator for calculating OD. All eligible patients were sorted by OD in the county of residence, and OD was divided into 4 categories (<2.9, 2.9–6.5, 6.5–8.4, and >8.4 oncologists per 100,000 population), so that each category of OD would have a similar number of individuals newly diagnosed with metastatic solid cancer or hematologic malignancies. We also captured age, race, sex, marital status, use of radiation, and MUA/HPSA designation. Some FIPS code areas were covering multiple MUA or HPSA IDs. The area was considered as MUA or HPSA if it had at least one MUA or HPSA IDs with designated status. Our primary outcome was overall survival measured from SEER diagnosis date until death censored at the end of 2014 (3 years follow-up).

Using the same ranges of OD identified by utilizing the SEER dataset, we plotted the OD for all FIPS codes on the map of mainland USA. We also plotted FIPS code areas with > 6.5 oncologists per 100,000 population designated as HPSA/MUA and FIPS code areas with < 2.9 oncologists per 100,000 population, which were not designated as HPSA or MUA, as areas with potentially misclassified MUA or HPSA status.

### Statistical analysis

Overall survival was estimated using the Kaplan-Meier method and compared between categories using a log-rank test. For multivariate analysis, we used Cox Proportional Hazard modeling with the primary site and histology as our stratification variable to study overall survival. Since MUA and HPSA designation were correlated with each other, a composite variable of MUA or HPSA status was included in the model as a covariate. Since MUA and HPSA designation also accounts for percent of the population below 100% federal poverty level and travel time to the nearest source of care [12,13], we indirectly controlled for socioeconomic and geographic differences in different FIPS codes.

We also calculated the proportion of counties with MUA or HPSA designation for each OD category and the percentage of oncologists working on a visa for each FIPS code area stratified by the OD category. Spearman’s rank-order correlation and Cochran- Armitage trend test measured the statistical significance of the difference in the proportion of counties. A p-value of less than 0.05 was considered significant. All analysis was performed using SAS 9.4 (SAS Institute, Cary, NC).

### Sensitivity analysis

We also performed sensitivity analysis by dividing FIPS codes in five OD categories (OD = 0, >0 to 3, >3 to 6, >6 to 9, and >9 oncologists per 100,000 population). The results were similar and reported in an online supplement.

## Results

A total of 68,699 patients were diagnosed with hematologic malignancies or metastatic solid cancers in 2011 within the SEER dataset. The mean age was 67.5 years. 39,177 (57%) were males, 51,475 (74.9%) had metastatic solid cancers and 17,224 (25.1%) had hematologic malignancies. Table 1 describes the baseline characteristics of patients treated in areas with different categories of OD. Patients were located within 609 US FIPS code areas, and 3,983 oncologists were identified to be working in these areas. The mean age of oncologists was 51.7 years, with 2,887 (72.5%) oncologists being male. A total of 247 (6.2%) oncologists were working on a visa. Nearly 64% (390 of 609) FIPS code areas had no oncologists. Out of these 25, FIPS codes had at least 35,000 population and had no oncologists.

**Table 1:** Baseline characteristics of patients treated in FIPS code areas with different oncologist density.

Oncologist per 100,000 population	< 2.9	2.9–6.5	6.5–8.4	> 8.4	P-value
**Number of patients**	17,150	16,761	16,846	17,942	
**Age** (mean)	67.0	67.8	67.7	67.8	< .0001
**Female sex** (%)	41.1	42.9	43.5	44.0	< .0001
**Race** (%)					< .0001
**White**	86.1	81.9	77.1	76.4	
**Black**	10.3	8.3	14.0	16.4	
**Other**	3.2	8.9	8.3	6.4	
**Unknown**	0.3	0.9	0.6	0.8	
**Married marital status** (%)	52.4	48.8	48.5	49.1	< .0001
**Use of radiation** (%)	21.1	20.9	18.6	19.9	< .0001
**Median survival** (months)	8	9	9	11	< .0001

The median survival of patients in counties with the lowest OD was significantly lower compared to counties with the highest OD (8 months vs. 11 months, p<0.0001). After controlling for confounders, compared to patients in areas with the highest category of OD (>8.4 oncologists per 100,000 population), patients in areas with 3^rd^, 2^nd^, and 1^st^ category (6.5–8.4, 2.9–6.5, <2.9 oncologists per 100,000 population respectively) of OD had worse overall survival (HR 1.03, 95%CI 1.01–1.06, p = 0.01; HR 1.07, 95%CI 1.05–1.1, p<0.001; HR 1.12, 95%CI 1.09–1.15, p<0.001 respectively). MUA or HPSA status had no impact on survival (HR 1.03, 95%CI 0.97–1.09, p = 0.3) (Table 2). These results remained statistically significant in the subgroup analysis of patients with hematologic malignancies and metastatic solid cancers and sensitivity analysis by dividing FIPS codes into five OD categories (S1–S3 Tables).

**Table 2:** Proportional hazard model for survival stratified by primary site and histology.

Variable	Hazard Ratio	95% Lower CI	95% Upper CI	P-value
**Oncologist per 100,000 population**				
** > 8.4**	Referent			
** 6.5–8.4**	1.03	1.01	1.06	0.01
** 2.9–6.5**	1.07	1.05	1.1	<0.001
** < 2.9**	1.12	1.09	1.15	<0.001
**MUA or HPSA status**	1.03	0.97	1.09	0.3
**Age**	1.02	1.02	1.02	<0.001
**Female sex**	0.87	0.86	0.89	<0.001
**Race**				
** White**	Referent			
** Black**	1.09	1.06	1.12	<0.001
** Other**	0.90	0.87	0.93	<0.001
** Unknown**	0.37	0.3	0.46	<0.001
**Marital status (married vs. other)**	0.85	0.83	0.86	<0.001
**Use of radiation**	0.76	0.74	0.78	<0.001

Within the SEER region, 93.3% (568 of 609) of FIPS code areas were designated as having MUA or HPSA status. There was no difference in the proportion of FIPS code areas with MUA or HPSA designation among the 4 OD category (93.2%, 95.4%, 90.3%, and 91.7% from 1st to 4rth category, p = 0.7 for trend, spearman correlation = -0.003, p = 0.9). Assessing MUA and HPSA status separately yielded similar results, with comparable proportions of FIPS code areas with MUA designation (85.1%, 88.6%, 83.9%, and 85.4% from 1st to 4rth OD category, p = 0.9 for trend, spearman correlation = 0.02, p = 0.7) and HPSA designation (83.7%, 80.7%, 87.1%, and 75% from 1st to 4rth OD category, p = 0.2 for trend, spearman correlation = -0.04, p = 0.3).

There was also no significant difference in the proportion of oncologists working on visas among the 4 OD categories (7.3%, 5.2%, 6.7%, and 6.1% from 1^st^ to 4^th^ category, p = 0.5 for trend, Spearman correlation = 0.001, p = 0.9). Additionally, there was no difference in the proportion of oncologists working on visas among FIPS codes designated MUA or HPSA compared to areas with no MUA or HPSA designation (6.3% v. 4.0% respectively, p = 0.6).

**Fig 1:**
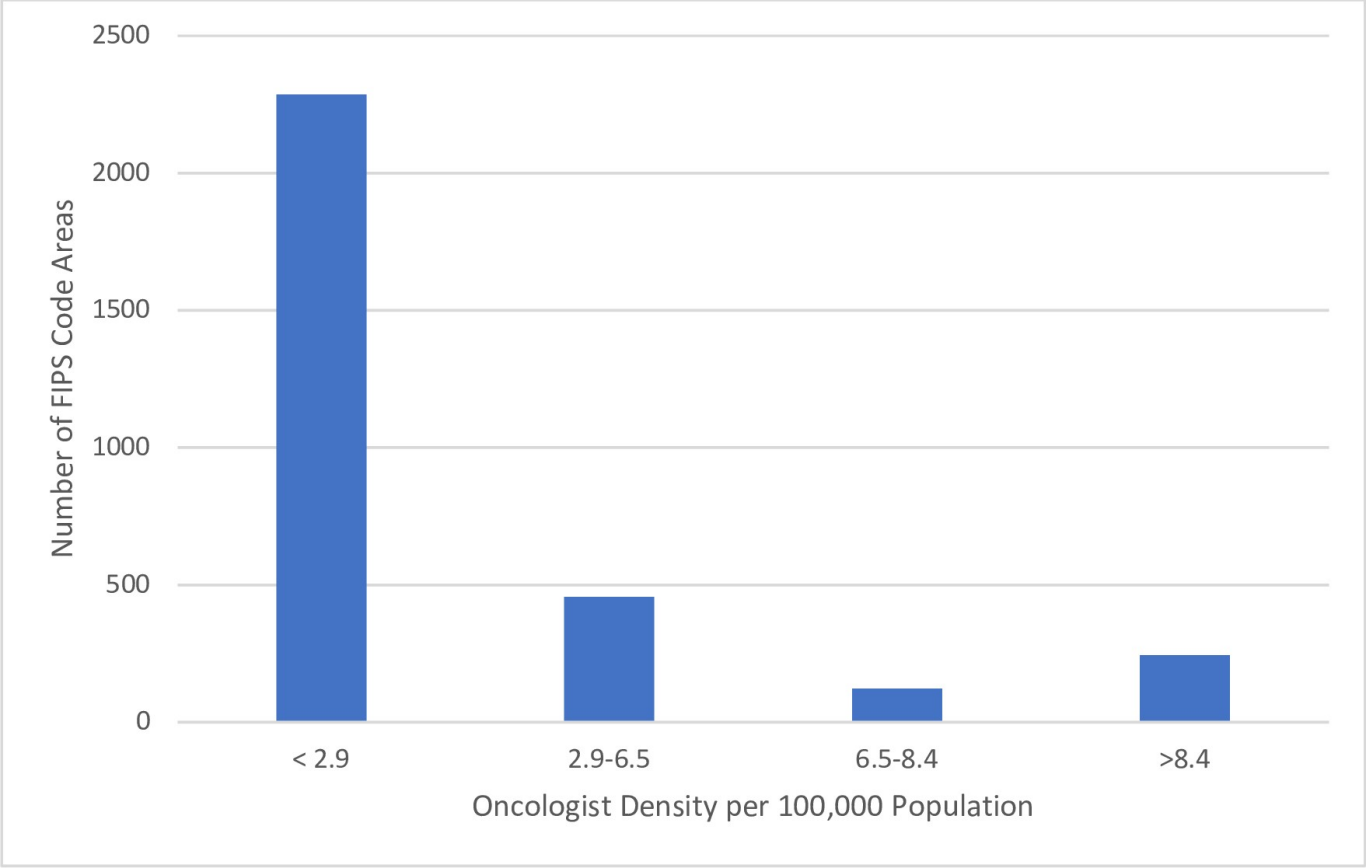
Distribution of oncologist density in different FIPS code areas in the United States.

Fig 1 shows the distribution of OD in all available FIPS codes in mainland USA (n = 3,108) using the same cutoffs for OD as above. Among counties with > 6.5 oncologist per 100,000 population (n = 366), 92.3% (n = 338) were designated as HPSA or MUA. Among counties with < 2.9 oncologists per 100,000 population (n = 2,284), 4.9% (n = 112) were not designated as HPSA or MUA, these represent counties with potentially misclassified underserved status in the context of oncologic care. Among 338 FIPS codes with > 6.5 oncologists per 100,000 population and designated HPSA or MUA status, 781 oncologists were working on visas. Among 112 FIPS codes with < 6.5 (Category 2.9–6.5 and <2.9) oncologists per 100,000 population and not designated as HPSA or MUA, only 1 oncologist was working on a visa.

## Discussion

While ASCO’s recent report predicted a significant shortage of oncologists [2], no prior studies show an association of outcomes with a density of oncologists per population. Our study shows that patients in areas with lower OD may correlate with worse overall survival. It may be helpful to increase the number of oncologists in areas with low OD to help reduce disparities in oncologic care in these areas.

Our study does not explain potential reasons for inferior OS among cancer patients living in areas with low OD. We did not have the ability to investigate the specifics of prognostic factors and treatment variables in our study population. However, our findings should be interpreted in the context of accumulating evidence that suggests increasing travel distance as a barrier to receiving appropriate cancer treatment. Lin et al. studied the impact of travel distance on receipt of adjuvant chemotherapy among 35,000 patients undergoing colon cancer resection. The authors demonstrated a low likelihood of receiving adjuvant therapy (odds ratio 0.36, p-value <0.01) for patients traveling >250 miles as compared to those with travel distances less than 12.5 miles [15]. Similar findings have been reported in patients receiving adjuvant radiation therapy for patients who have prostate cancer with high-risk pathologic features at radical prostatectomy [16]. Moreover, Aneja et al. showed that counties with at least 1 radiation oncologist had improved prostate cancer-specific mortality as compared to those with no radiation oncologists [17]. In surgical oncology, Odisho et al. studied the impact of urologist density on survival from urologic cancers, demonstrating a significant reduction in cancer-specific mortality for kidney, prostate, and bladder cancers in counties with 1 or more urologists as compared to none [18]. Our study possibly adds to the growing body of evidence showing the impact of non-PCP physicians’ density on outcomes.

In 2004, the Conrad-30 program was amended to allow states to sponsor waivers of home residency requirements of IMG specialists (e.g., Oncologist) on a J1 visa to work in underserved areas [19]. Physician scarcity area (PSA) designation, which accounted for a shortage of specialists, was available to identify areas with a specialist shortage. However, the Medicare program, which defined PSA expired in 2008, and PSA designation is no longer available to be used by states [20]. Hence, HPSA and MUA designations are used by states to place physicians, including specialists, in underserved areas. Our study shows that MUA and HPSA designations are not concordant with OD. In fact, 91.7% of counties in the SEER region with the highest oncologist density (>8.4 per 100,000 population) were classified as MUA or HPSA areas. Hence, placing specialists (i.e., oncologists) in areas with MUA or HPSA designation may not reduce variation in OD and may further exacerbate disparities in OD.

In the Conrad-30 program, each state has a fixed number of waivers (30 per state) every year regardless of the total population or the total number of physicians working in the state. Certain states receive greater than 30 applications every year, and every spot is assigned to PCP applicants [21]. In contrast, other states receive less than 30 applications every year, and greater than half of them are filled by specialists [22]. Since the number of spots is fixed, the discrepancy in applications received by states and priority given to PCP applicants creates an imbalance in the placement of specialists in relevant underserved areas, which varies depending on the size, population, and physician density of the state.

Our study shows that there is no difference in the proportion of oncologists working on visas among areas in 4 categories of OD. Even in areas with the lowest OD (<2.9 oncologists per 100,000 population), greater than 90% of oncologists are US citizens or permanent residents. This shows an opportunity to create designated spots for oncology trainees on a J1 visa to work in areas with low OD after completion of the training. Per our communication with the education commission for foreign medical graduates (ECFMG), there were 199, 200, and 229 hematology-oncology fellows in training on J1 visa in 2015, 2016, and 2017 respectively. They would graduate over a period of 3 years (S4 Table). While some of these may choose to go back to their home countries, many would likely be willing to work in underserved areas in the USA for 3 years. Hence, amending Conrad-30 to create designated spots for specialists like oncologists to work in relevant underserved areas may help to reduce healthcare disparities in specialty care.

Our study was only designed to assess the impact of medical oncologists’ density on overall survival. We acknowledge that cancer treatment and its outcome involve multidisciplinary input, which includes access to subspecialists (eg., Colorectal surgery), availability of advanced imaging/screening techniques, accessibility to tertiary centers with clinical trial options, and awareness among the primary care physicians about cancer care. For example, the patients with CNS metastasis and primary CNS cancers may benefit from treatment from neuro-oncologists, and patients with early-stage solid cancers often require other treatment modalities, including curative surgeries or definitively chemo-radiation hence we have attempted to exclude some of these patients to decrease the confounding effect. Due to the limitation of the data, we could not control for additional disease-related confounders (molecular markers), treatment-related confounders (specific treatment received by each patient), and socioeconomic differences (income, insurance coverage, and education).

## Conclusion

Patients residing in areas with lower OD may have worse overall survival following a cancer diagnosis. MUA or HPSA designation is not concordant with OD in different FIPS code areas and probably not associated with survival. In the current format, the Conrad-30 program is not designed to promote the placement of oncologists on a visa in areas with low OD. Our health care policies’ effectiveness on reducing disparities in oncologic care depends on improved data and a more tailored solution beyond the current Conrad-30 program as similar to personalized medicine in cancer care. Amending the Conrad-30 program to include designated spots for specialists like oncologists and improving targeting beyond MUA/HPSA designation is likely to increase the oncology workforce in the most underserved areas.

## Supporting information

S1 Table Proportional hazard model for survival stratified by primary site and histology for patients with metastatic solid tumors. (DOCX)

S2 Table Proportional hazard model for survival stratified by primary site and histology for patients with hematologic malignancies. (DOCX)

S3 Table Proportional hazard model for survival stratified by primary site and histology. (DOCX)

S4 Table Hematology-oncology fellows in training on J1 visa in 2015, 2016 and 2017. (DOCX)

S1 File Supplementary methods and results. (DOCX)
