# Supplementary material for: Overall survival based on oncologist density in the United States: A retrospective cohort study
Source: PLoS One. 2021 May 12;16(5):e0250894. doi: 10.1371/journal.pone.0250894 (PMC8115849; doi:10.1371/journal.pone.0250894)
Supplement: S1 Table — (DOCX) [file pone.0250894.s001.docx]

Supplementary Table 1: Proportional hazard model for survival stratified by primary site and histology for patients with metastatic solid tumors

| **Variable** | **Hazard Ratio** | **95% Lower CI** | **95% Upper CI** | **P Value** |
| --- | --- | --- | --- | --- |
| **Oncologist per 100,000 population** |  |  |  |  |
| **>8.4** | Referent |  |  |  |
| **6.5-8.4** | 1.02 | 0.99 | 1.05 | 0.15 |
| **2.9-6.5** | 1.06 | 1.03 | 1.09 | <0.001 |
| **<2.9** | 1.10 | 1.07 | 1.13 | <0.001 |
| **MUA or HPSA Status** | 1.04 | 0.98 | 1.11 | 0.16 |
| **Age** | 1.02 | 1.02 | 1.02 | <0.001 |
| **Female sex** | 0.88 | 0.86 | 0.90 | <0.001 |
| **Race** |  |  |  |  |
| **White** | Referent |  |  |  |
| **Black** | 1.08 | 1.05 | 1.11 | <0.001 |
| **Other** | 0.88 | 0.85 | 0.92 | <0.001 |
| **Unknown** | 0.53 | 0.41 | 0.67 | <0.001 |
| **Marital status (married vs. other)** | 0.86 | 0.84 | 0.87 | <0.001 |
| **Use of radiation** | 0.74 | 0.72 | 0.76 | <0.001 |
