# Supplementary material for: Overall survival based on oncologist density in the United States: A retrospective cohort study
Source: PLoS One. 2021 May 12;16(5):e0250894. doi: 10.1371/journal.pone.0250894 (PMC8115849; doi:10.1371/journal.pone.0250894)
Supplement: S2 Table — (DOCX) [file pone.0250894.s002.docx]

Supplementary Table 2: Proportional hazard model for survival stratified by primary site and histology for patients with hematologic malignancies

| **Variable** | **Hazard Ratio** | **95 % Lower CI** | **95% Upper CI** | **P Value** |
| --- | --- | --- | --- | --- |
| **Oncologist per 100,000 population** |  |  |  |  |
| **>8.4** | Referent |  |  |  |
| **6.5-8.4** | 1.10 | 1.04 | 1.17 | 0.002 |
| **2.9-6.5** | 1.13 | 1.06 | 1.20 | <0.001 |
| **<2.9** | 1.20 | 1.13 | 1.27 | <0.001 |
| **MUA or HPSA Status** | 0.94 | 0.82 | 1.07 | 0.35 |
| **Age** | 1.04 | 1.04 | 1.05 | <0.001 |
| **Female Sex** | 0.84 | 0.80 | 0.88 | <0.001 |
| **Race** |  |  |  |  |
| **White** | Referent |  |  |  |
| **Black** | 1.15 | 1.07 | 1.23 | <.0001 |
| **Other** | 1.02 | 0.93 | 1.11 | 0.74 |
| **Unknown** | 0.19 | 0.12 | 0.30 | <0.001 |
| **Marital status (married vs. other)** | 0.79 | 0.76 | 0.83 | <.0001 |
| **Use of Radiation** | 1.09 | 1.00 | 1.19 | 0.04 |
