# Supplementary material for: Overall survival based on oncologist density in the United States: A retrospective cohort study
Source: PLoS One. 2021 May 12;16(5):e0250894. doi: 10.1371/journal.pone.0250894 (PMC8115849; doi:10.1371/journal.pone.0250894)
Supplement: S4 Table — (DOCX) [file pone.0250894.s004.docx]

Supplementary Table 4: Hematology-oncology fellows in training on J1 visa in 2015, 2016 and 2017

| **Internal Medicine Subspecialty** | **2015** | **2016** | **2017** |
| --- | --- | --- | --- |
| **Hematology** | 2 | 3 | 1 |
| **Hematology and Medical Oncology** | 0 | 0 | 1 |
| **Hematology and Oncology** | 197 | 199 | 226 |
| **Hematology/Oncology- Experimental Therapy** | 2 | 1 | 2 |
| **Medical Oncology** | 0 | 0 | 0 |
| **Total** | 201 | 203 | 230 |

Data was obtained from Education Commission for Foreign Medical Graduates (ECFMG)
