# Supplementary material for: Overall survival based on oncologist density in the United States: A retrospective cohort study
Source: PLoS One. 2021 May 12;16(5):e0250894. doi: 10.1371/journal.pone.0250894 (PMC8115849; doi:10.1371/journal.pone.0250894)
Supplement: S1 File — (DOCX) [file pone.0250894.s005.docx]

**Supplementary methods and results**

We performed sensitivity analysis by dividing FIPS codes in five OD categories (OD = 0, >0 to 3, >3 to 6, >6 to 9, and >9 oncologists per 100K population).

After controlling for confounders, compared to patients in FIPS code areas with highest category of OD (>9 oncologists per 100K population), patients in areas with 4^th^, 3^rd^, 2^nd^, and 1^st^ category (>6 to 9, >3 to 6, >0 to 3, and 0 oncologists per 100K population respectively) of OD had worse overall survival (HR 1.04, 95%CI 1.01-1.06, p=0.007; HR 1.08, 95%CI 1.05-1.11, p<0.001; HR 1.12, 95%CI 1.09-1.16, p<0.001; HR 1.13, 95%CI 1.10-1.17, p<0.001 respectively). MUA or HPSA status had no impact on survival (HR 1.03, 95%CI 0.98-1.09, p=0.27). (Table S3)

There was no difference in the proportion of FIPS code areas with MUA or HPSA designation among the 5 OD category (93.6%, 91.2%, 94.5%, 90.6% and 94.4% from 1^st^ to 5^th^ category, p=0.8 for trend, spearman correlation= -0.01, p=0.72). Assessing MUA and HPSA status separately yielded similar results, with comparable proportions of FIPS code areas with MUA designation (84.9%, 87.7%, 87.7%, 81.1% and 91.7% from 1^st^ to 5^th^ OD category, p=0.57 for trend, spearman correlation= 0.02, p=0.55) and HPSA designation (84.6%, 77.2%, 80.8%, 79.3% and 80.6% from 1^st^ to 5^th^ OD category, p=0.21 for trend, spearman correlation= -0.06, p=0.14).
